# Effectiveness of combined extracorporeal shock-wave therapy and hyaluronic acid injections for patients with shoulder pain due to rotator cuff tendinopathy: a person-centered approach with a focus on gender differences to treatment response

**DOI:** 10.1186/s12891-022-05819-3

**Published:** 2022-09-15

**Authors:** Raffaello Pellegrino, Angelo Di Iorio, Fabrizio Brindisino, Teresa Paolucci, Antimo Moretti, Giovanni Iolascon

**Affiliations:** 1 Antalgic Mini-invasive and Rehab-Outpatients Unit, Department of Innovative Technologies in Medicine & Dentistry, University “G. d’Annunzio”, 66100 Chieti-Pescara, Italy; 2 Department of Scientific Research, Campus Ludes, Off-Campus Semmelweis University, 6912 Lugano, Pazzallo Switzerland; 3 Department of Medicine and Health Science “Vincenzo Tiberio”, University of Molise c/o Cardarelli Hospital, C/da Tappino, 86100 Campobasso, Italy; 4 Physical Medicine and Rehabilitation, Department of Oral Medical Science and Biotechnology, University G D’Annunzio, 66100 Chieti-Pescara, Italy; 5 Department of Medical and Surgical Specialties and Dentistry, University of Campania, “Luigi Vanvitelli”, 80138 Naples, Italy

**Keywords:** Extracorporeal shock wave (ESWT), Hyaluronic acid, Linear Mixed model, Peritendinous injection, Rotator cuff (RC), Shock wave therapy, Shoulder pain, SPADI, NRS, Tendinopathy

## Abstract

**Background:**

Rotator cuff (RC) tendinopathy is a common shoulder pain condition. Extracorporeal shockwave therapy (ESWT) and hyaluronic acid peritendinous injection are viable treatment options for RC tendinopathy.

The aim of this study is to evaluate the response in two different therapeutic rehabilitative approaches, the combined treatment ESWT plus hyaluronic acid injections (E + Hy) compared to ESWT alone (ESWT-al), in a cohort of patients with RC tendinopathy according to gender differences.

**Methods:**

This is a retrospective longitudinal cohort study of patients with painful RC tendinopathy. Patients that had received a clinical evaluation, a shoulder ultra sound examination, as well as the Shoulder Pain and Disability Index (SPADI) questionnaire, and the Numerical Rating Scale (NRS) for pain at baseline, 1-month (T1) and 2-month follow-ups (T2) were included.

**Results:**

Medical records of 53 patients were analyzed. In the comparison between baseline to T1 and similarly from baseline to T2, a statistically significant reduction has been reported in the NRS (*p* < 0.001) and in the SPADI (*p* < 0.001) in the entire study group. At T1, patients in the E + Hy compared to ESWT-al group, showed a slight but statistically significant reductions in both NRS and SPADI score, while these changes were more evident at T2 (*p* < 0.001). Interestingly, a gender dimorphism in NRS and in SPADI was found, with female patients that apparently responded better to the combined E + Hy compared to ESWT-al approach.

**Conclusion:**

This retrospective cohort study suggests that the combination of ESWT plus HyA injections seems to be more effective than ESWT alone in RC tendinopathy in both genders. Moreover, in ESWT alone treatment, male patients reported better outcomes compared to females. However, further randomized controlled trials should be structured to confirm and enforce these conclusions.

**Supplementary Information:**

The online version contains supplementary material available at 10.1186/s12891-022-05819-3.

## Introduction

Shoulder pain is a common clinical complaint, with a point prevalence reported ranging from 7 to 26% in the general population, with a lifetime prevalence of up to 67% [1]. The incidence of shoulder pain is estimated to be 19 per 1000 person-years, highest in women over 45 years [2].

Among shoulder pain problems, rotator cuff (RC) pathology is one of the more common conditions for which patients seek treatment [3]; in particular RC tendinopathy refers to pain and weakness, most commonly experienced with movements of shoulder external rotation and elevation, as a consequence of excessive load on RC tissues [4]. Although load alteration is implicated as the major factor associated with clinical onset of this condition, its pathogenesis is likely to be multifactorial, and this has led to several management strategies, from conservatives to mini-invasive [5] and surgical treatments [6].

In the context of the surgical approaches, several arthroscopic procedures are proposed for rotator cuff tears. Among them a tear completion repair (TCR) technique, in the mid-term as in the long-term follow-up, showed good outcomes in terms of functional and strength recovery, patient satisfaction, and resolution of pain [7, 8].

General consensus showed that tendinopathy, at least in the chronic stage, is mainly a degenerative condition, inflammation plays a minor role [9]. As matter of fact this concept has led to a shift from treatments that target inflammation towards treatment options that promote regeneration [10]. One of these treatments is extracorporeal shockwave therapy (ESWT), a physical therapy modality that generates three-dimensional pressure pulses, lasting microseconds and reaching peek pressures of 35–120 MPa; the effects of this intervention depend on intensity, pulse cycle and shockwave (SW) modality [11].

Several possible mechanisms have been proposed for the effect of ESWT, including overstimulation of nociceptor that produces an analgesic effect, or microlesions of the tendon tissue by the physical effects of the sound waves (i.e. radial shock wave) ending in the promotion of a healing process of the tendon [12]. The success rate ranged from 60 to 90%, and the complications were low and negligible [13]. As a side effect, the treatment itself could be unpleasant or painful, and can cause redness and superficial hematomata on the skin [14]. Due to the pain during ESWT application, the patient might develop a vago-vasal reaction [15]. EWST could trigger a procedural discomfort or pain, therefore could induce physician to modulate the intensity of administered shockwaves, lasting in a reduced efficacy of the treatment.

Numerous clinical studies have demonstrated the therapeutic capacity of exogenous peritendinous hyaluronic acid (HyA) injection in reducing pain, improving function and reducing tendon rubbing in pre-insertion areas in the course of the different tendinopathies (e.g. affecting RC, epicondyle, patellar and Achilles tendons), tendon injuries and tendon reparative post-surgery [16]. Preclinical in vitro and in vivo studies have shown the ability of exogenous peritendinous HyA to perform an inhibitory effect on the fibroblast activity and a shift from M1, pro-inflammatory state, toward M2 phenotype (anti-inflammatory) during tendinopathy [17].

An overall difference was reported in disability recovery, and response to shoulder pain treatment between men and women [18]. In particular, female patients had a lower functional recovery following a RC repair [19], as well as lower improvement in the shoulder functional and self-reported disability measures after RC surgery [20]. Overall, taking into account only surgical approaches, women appear to be more disabled both prior to and after RC treatment, and reported higher level of discomfort in comparison with men. Several determinants are argued to explain and/or to modulate those differences, such as the pre-surgical expectation, tendon injury grade, comorbidity, polypharmacotherapy, timing and gender-specific rehabilitative program, and sex-gender interaction in specific pain response differences [18, 21].

The objectives of this study were to investigate the effectiveness in terms of pain and disability of ESWT plus Hyaluronic Acid injections (E + Hy) compared to ESWT-alone (ESWT-al) in patients with painful RC tendinopathy, as well as to compare the overall treatment response between male and female patients.

## Methods

We performed a retrospective longitudinal cohort study including medical records of patients with painful RC tendinopathy, referred at the Chiparo Physical Medicine and Rehabilitation outpatient in Lecce between July 2021 and December 2021. The study was planned at the Clinical Research Department of the Ludes Campus in Lugano (CH), and complies with the STROBE guidelines of the Consort Statement for the reporting of observational studies.

To be eligible, patients had to be between 30 and 65 years old, with shoulder pain intensity at the Numerical Rating Scale (NRS) of at least 5, and had to be negative on screening for other sources of pain outside the shoulder (e.g., cervical spine). The diagnosis of RC had to be confirmed by US evaluation showing structural inhomogeneity and altered tendon thickness [22]. Patients with the US-scan-confirmed tendon injury or rupture, clinical history of shoulder surgery or previous shoulder injections, specific rehabilitation treatments within 2 months prior to access to the outpatients were excluded. Additional exclusion criteria were patients with fibromyalgia, enthesitis due to seronegative arthritis, rheumatoid arthritis, and cancer; systemic inflammatory disease, osteomyelitis, active infection or history of chronic infection in the treatment area, neurological disorders or vascular insufficiencies, nerve entrapment syndrome, disturbance of coagulation or ongoing anticoagulation therapy [19].

The study was developed following the Good Clinical Practice (GCP) guidelines. It was conducted within the ethical principles outlined in the Declaration of Helsinki, and with the procedures defined by the ISO 9001-2015 standards for “Research and experimentation”. Written informed consent to provide information included in personal medical records were obtained from all participants.

### Outcome measures

Clinical data (i.e., comorbidities, drug intake, potential risk factors for adverse drug reactions), US examination of the shoulder, as well as a disability evaluation assessed by the Shoulder Pain and Disability Index (SPADI) questionnaire were collected [23, 24]. The SPADI was a questionnaire, a self-completed district assessment tool, commonly used to assess shoulder musculoskeletal disorders. The questionnaire consists of 13 items divided into two modules designed to assess shoulder pain (5 items) and disability (8 items). The answers are indicated on a visual analogue scale where 0 is no pain/ no difficulty and 10 is worst imaginable pain/so difficult it requires help. The items are summed and converted to a total score out of 100. Pain was assessed by the use of the Numerical Rating Scale (NRS) [25] that variates between 0 and 10 with 0 indicating no pain and 10 indicating the worst possible pain. Outcomes measures were available at baseline (T0), at day 30 (T1) during the intervention, and at the end of the study, at day 60 (T2).

### Extracorporeal shockwave therapy

Both groups (E + Hy and ESWT-al) received three sessions of ESWT (at day 1, day 7, day 14), administered by the same operator. For each single therapeutic session with ESWT, 2000 pulses emitted by focused shock wave generator (piezoclast-EMS ESWT) with a range of energy flux density between 0.11–0.28 mmJ/mm2 and a frequency between 6 and 8 Hz were administered. The treatment was administered regularly, and titrated up to remain perceived as ‘strong but comfortable’ during use. Patients were seated, and the affected shoulder was positioned in adduction and internal rotation with the elbow in flexion and the palm resting on the back during the execution of the first 1000 impulses and then upper limb in neutral position of shoulder rotation with arm along the trunk for the next 1000 impulses.

### Peritendinous hyaluronic acid injection

Only E + Hy, 4 days after the last therapeutic ESWT session, received HyA in the *peritendinous area* of the supraspinatus using real-time US guide infiltration (SonoSite M-Turbo ultrasound system with a 6–15 Mhz linear probe). Peritendinous infiltration with HyA was administered twice more, at day 18 and 25 from last ESWT session. Injection was done using a pre-filled syringe of 20 mg in 2 ml sodium salt of linear HyA with molecular weight 500–730 kDa, with 21G 40 mm needles. No anesthetic drugs were administered before and after the treatment. During the procedure, patients were seated, and the affected shoulder was positioned in adduction and internal rotation with the elbow in flexion and the palm resting on the back. The skin was disinfected with a chlorhexidine wipe.

### Rehabilitation program after intervention

After intervention, both study groups underwent a 45 minutes/session, twice a week, for 6 weeks period of rehabilitation exercise with physiotherapist, consisting in a standardized protocol including: a) Protraction of the scapula; b) closed chain scapulo-thoracic contraction; c) closed chain protraction of the scapula; d) wall slide; e) upward rotation of the scapula; f) retraction of the scapula [26].

### Statistical analysis

Data were reported as mean ± standard error (S.E.) for continuous variables and as absolute number and percentage for dichotomous variables; differences between groups were assessed with analysis of variance and chi-square test, respectively. To assess the variation of SPADI-score, and NRS during the study, Linear Mixed Models were applied [27]. Intercept and Time had a random component. The advantage of this approach is that it increases the precision of the estimate by using all available information concerning performance and, at the same time, allow for handling missing data, and had a more powerful modelling of the analysis.

In the both LMMs were considered the: two treatments, with ESWT-al as the reference group; the three times of the study, with baseline as the reference; the interaction between time and treatment; sex effect, with female as the reference; the interaction between time and sex to assess gender differences in the response to treatment; shoulder side treated, with left as the reference; and lastly age as a continuous variable. Data were analyzed with SAS-software (rel. 9.4), and *p*-value for differences was considered statistically significant for a value less or equal to 0.05.

## Results

Medical records of 53 patients, 22 (41.5%) males, and 31 (58.5%) females, were collected, including 33 patients in the combined treatment group (E + Hy), and 20 patients in the ESWT-al group. Mean age for study cohort was 53.0 ± 7.4 (range 40–65); no statistically significant differences were found for age according to sex (52.1 ± 7.6 in male, 53.7 ± 7.3 in female; *p*-value = 0.45), whereas a statistically significant difference was found for age according to treatment (54.6 ± 7.6 and 50.4 ± 6.4 in E + Hy and ESWT-al respectively; *p*-value = 0.05). Twenty patients (37.7%) reported pain at the left shoulder, whereas 33 on the right shoulder without statistically significant differences between the two treatment groups (*p*-value = 0.75). The mean NRS was 6.6 ± 1.0, and the mean SPADI-score was 47.8 ± 10.4 in the study cohort. No statistically significant differences in both outcome measures were found according to sex (NRS 6.6 ± 0.9 and 6.8 ± 1.1; *p*-value = 0.46 in E + Hy and in ESWT-al respectively; SPADI-score 49.7 ± 10.9 and 47.8 ± 9.0; *p*-value =0.10 in E + Hy and in ESWT-al respectively). In Tables 1 and 2 were reported the variations in the NRS and in SPADI according to time of the study, type of treatment, sex, side of shoulder pain, and age. Statistically significant reduction was demonstrated for NRS between baseline and Follow-up-1 and similarly from baseline to Follow-up-2 (− 1.98 ± 0.34; *p*-value< 0.001; − 1.87 ± 0.34; *p*-value < 0.001, for Follow-up-1 and Follow-up-2, respectively). E + Hy group compared to ESWT-al showed a slight but significant reduction in the NRS at the follow-up-1 (− 0.67 ± 0.34; *p*-value = 0.05), and this change was more evident at follow-up-2 (− 1.85 ± 0.39; *p*-value< 0.001). Taking into account the NRS change according to sex, male patients reported statistically significant pain relief at the follow-up-2 compared to female sex (− 1.45 ± 0.38; *p*-value< 0.001). Lastly within person variance in the NRS in function of time was 88.7%.

**Table 1:** Mixed Linear Model; NRS variation according to time of the study and treatment

Effetto	Estimate	S.E.	Pr > |t|
Intercept	6.10	1.12	< 0.001
Treatment E + Hy	−0.32	0.38	0.41
Treatment ESWT-al (reference group)			
Time Follow-up 2	−1.87	0.34	< 0.001
Time Follow-up 1	−1.98	0.34	< 0.001
Baseline (reference group)			
Time Follow-up 2 Treatment E + Hy	−1.85	0.39	< 0.001
Time Follow-up 2 Treatment ESWT-al (reference group)			
Time Follow-up 1 Treatment E + Hy	−0.67	0.34	0.05
Time Follow-up 1 Treatment ESWT-al (reference group)			
Baseline (reference group)			
Sex Male	0.65	0.37	0.10
Sex Female (reference group)			
Time Follow-up 2 Sex Male	−1.45	0.38	< 0.001
Time Follow-up 2 Sex Female (reference group)			
Time Follow-up 1 Sex Male	0.08	0.38	0.85
Time Follow-up 1 Sex Female (reference group)			
Shoulder pain side right	−0.70	0.32	0.03
Shoulder pain side left (reference group)			
Age (yy)	0.01	0.02	0.44

**Table 2:** Mixed Linear Model; SPADI variation according to time of the study and treatment

Effetto	Estimate	S.E.	Pr > |t|
Intercept	36.85	10.17	< 0.001
Treatment E + Hy	3.85	3.33	0.25
Treatment ESWT-al (reference group)			
Time Follow-up 2	−18.13	2.59	< 0.001
Time Follow-up 1	−16.62	2.58	< 0.001
Baseline (reference group)			
Time Follow-up 2 Treatment E + Hy	−13.22	2.96	< 0.001
Time Follow-up 2 Treatment ESWT-al (reference group)			
Time Follow-up 1 Treatment E + Hy	−10.43	2.95	< 0.001
Time Follow-up 1 Treatment ESWT-al (reference group)			
Baseline (reference group)			
Sex Male	3.87	3.23	0.24
Sex Female (reference group)			
Time Follow-up 2 Sex Male	−5.73	2.95	0.05
Time Follow-up 2 Sex Female (reference group)			
Time Follow-up 1 Sex Male	3.49	2.92	0.24
Time Follow-up 1 Sex Female (reference group)			
Shoulder pain side right	−4.90	2.90	0.10
Shoulder pain side left (reference group)			
Age (yy)	0.19	0.20	0.34

For SPADI statistically significant changes in follow-up-1 (− 16.62 ± 2.58; *p*-value< 0.001) and in follow-up-2 (− 18.13 ± 2.59; *p*-value< 0.001) compared to baseline were reported in the study cohort, independently from the treatment group. According to time points and treatment group, a multiplicative effect for the interaction was found, as matter of fact that mean SPADI-score in E + Hy group significantly decreased from baseline at both follow-up-1 and follow-up-2, (− 10.43 ± 2.95; *p*-value < 0.001; − 13.22 ± 2.96; *p*-value< 0.001, respectively) compared to ESWT-al group.

**Fig. 1:**
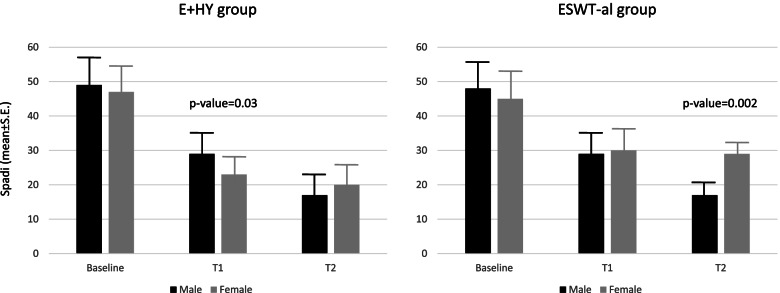
Average SPADI variation across time points of the study in the E + Hy (**A**) and in the ESWT-al (**B**) treatment groups. Male sex was reported in black bars, whereas female in grey bars. Post estimate specific time points *p*-value was reported when statistically significant

Moreover, statistically significant changes were found for SPADI only in male patients at the follow-up-2 (5.73 ± 2.95; *p*-value = 0.05). Lastly within person variance in the SPADI in function of time was 95%. To verify if a gender difference in terms of NRS and in SPADI changes could be attributable to the different type of intervention, the same approach with Mixed-Model was applied and analyses were stratified according to treatment. In male patients a similar response through times of the study according to different therapeutical approach could be found. Interestingly, female patients apparently respond better to a combined E + Hy compared to ESWT-al approach (Fig. 1A and B).

**Fig. 2:**
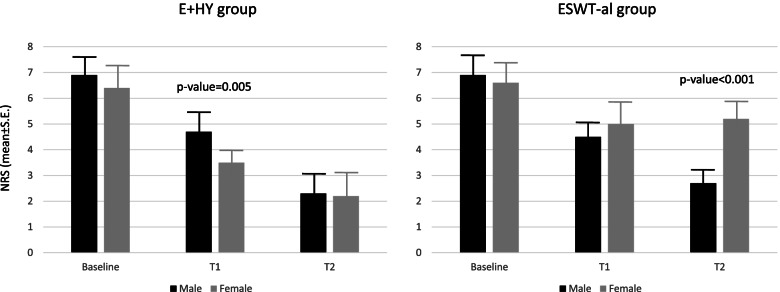
Average NRS variation across time points of the study in the E + Hy (**A**) and in the ESWT-al (**B**) treatment groups. Male sex was reported in black bars, whereas female in grey bars. Post estimate specific time points p-value was reported when statistically significant

As matter of fact, females of the E + Hy group versus those of ESWT-al group reported statistically significant lower scores at SPADI at both follow-up-1 and follow-up-2 (− 15.21 ± 3.35; *p*-value< 0.001; and − 19.45 ± 3.35; *p*-value< 0.001). According to the NRS, female patients of the E + Hy group reported statistically significant pain relief at follow-up-1, while both genders reached similar results at follow-up-2. In the ESWT-al group, male patients reported lower pain intensity at follow-up-1, and the trend of this change was confirmed also in follow-up-2 (Fig. 2A and B). No statistically significant difference was reported in self-reported pain (*p*-value = 0.08), between sex, in the same treatment group at follow-up-1. At follow-up-2, only male patients reported pain relief, while female patients did not, those differences in NRS between sexes become statistically significant (*p*-value< 0.001). We also performed a stratified analysis only on female, to compare the variation trend in NRS and in SPADI according to treatment approach, that showed a multiplicative effect (statistically significant time for treatment interaction) for pain and disability of the shoulder (Supplementary Tables 1 and 2). Lastly no serious adverse events were recorded and peritendinous injections of HyA as well as ESWT had been well tolerated by all patients.

## Discussion

This retrospective longitudinal observational study is aimed to investigate the effectiveness of two different rehabilitation protocols (i.e., HyA plus EWST versus EWST-alone) also considering potential gender-difference in the treatment response in a cohort of patients with RC tendinopathy. Our findings suggest that E + Hy results in better and faster response compared to EWST-al in terms of pain relief and disability reduction. Also, these results were maintained at the follow-ups with a further symptomatic improvement in the E + Hy group. According to literature, peritendinous injections of HyA seem to be effective in reducing pain and to improve functions in patients with RC tendinopathy [28, 29], although few evidence support this intervention.

Hyaluronic acid enhances the fibroblasts activity, including their adhesivity, extracellular matrix synthesis, and proliferation, although its effect on the biomechanical properties of tendon is still not clarified [30–32]. Our results confirmed previous reports who evaluated the effectiveness of US-guided HyA injections in patients with supraspinatus tendinopathy, reporting symptoms and disability improvement compared with placebo, up to 9-month follow-up [31]. Also, Frizziero et al. did not reported significant differences between HyA injections and ESWT in terms of safety and efficacy [33]. Then, HyA injections have just showed promising results in the treatment of tendinopathies with no side effects respect to glucocorticoids [13]. In our study, no serious adverse events were recorded and the interventions (i.e., peritendinous injections of HyA and ESWT) were well tolerated by all patients. Other researchers indicated that a cycle of 3 injections of 500–730 kDa HyA may determine long-term decrease in pain intensity in patients affected by chronic tendinopathies non-responders to conventional therapies until 56 days at follow-up [34]. Furthermore, in our study, we observed that pain continues to significantly decrease in the E + Hy group versus ESWT-al group, thus supporting the hypothesis of a stabilizing and reinforcing role in the resolution of pain played by the combination with the EWST [12]. Considering also the results of Galasso et al. who detected a remarkable short-term efficacy in functional shoulder recovery in patients receiving ESWT [35], we could hypothesize in our study, a first joint action of E + Hy and then, a maintenance of the clinical and functional benefits thanks to the HyA injections. In fact, according to the literature, ESWT produces repeated microtrauma that could stimulate the ingrowth of neovascularization associated with the up-regulation of angiogenic and osteogenic growth factors [12], but also, a pain modulation (i.e., hyperstimulation of nociceptors, gate-control theory) as well as tendon remodeling, causing fibroblast stimulation and vasodilation [36]. However, more research is needed to fully understand the underlying biological mechanisms to better understand the gender differences in terms of pain relief and disability reduction in favor of a more consistent positive response in males than females reported in our study. In fact, the available literature about this topic is still debated. As confirmed by some authors, no significant gender difference during ESWT was demonstrated [37]. On the other hand, Notarnicola et al. showed as male gender is a prognostic factor for improvement in pain and function during and after ESWT [38]. A gender role appears to influence response to pain, considering that male patients had higher withstanding pain, instead female patients showed higher pain sensitivity [39]. Then, for future studies, a shoulder tendinopathy ESWT protocol with greater intensity and power may be desirable for better maintenance of follow-up results in females. Our results, in fact, underlined as female patients respond better to a combined HyA plus ESWT compared to ESWT alone; instead, in the ESWT group male patients reported lower level of pain at T1, and the trend of the reduction was confirmed at T2 in comparison of female patients that showed a lower reduction in pain and shoulder function along treatment and observational times.

## Limitations

Among limitations to be accounted, the main relevant one deals with the retrospective study design that might be affected by several bias (e.g. selection bias). For example, as a retrospective study the allocation of patients in the two groups did not follow a randomization procedure; patients were assigned to one of the two groups according to the information contained in the medical records. Therefore, a healthy worker effect could be introduced; as matter of fact, those patients who participate in the E + Hy group represent subjects that are particularly interested in their performance and in their autonomy in self-care and could have more expectations for treatment success. The lack of an experimental design could not enable us to control for potential confounding factors (i.e. the work intensity, the adherence to rehab program, ESWD intensity and power variations according to gender) that could modulate and influence the performance, and the rehab process itself. We try in the analytic approach to compensate through a multivariate statistical analysis for potential confounding factors, as age, sex or side of the treated shoulder, but this approach could not be considered exhaustive. Synthetically, the external validity of this study is at least questionable, but the statistically significant differences found, and the biological plausibility of the results, could represent at least the basis to design a randomized clinical trial focused on the comparison between different approaches in the treatment of shoulder pain.

### Conclusion

Our study supports the hypothesis that a multimodal approach might provide additional benefits without safety concerns in patients with RC tendinopathy independently of gender. In particular, our retrospective investigation showed the effectiveness of combined intervention of ESWT and HyA injections over ESWT alone in subjects affected by RC tendinopathy in both men and women. However, in those receiving ESWT alone, men reported higher benefits in terms of pain relief compared to women. Despite these encouraging findings, randomized controlled trials are required to prove the efficacy of the proposed approach in patients with RC tendinopathy, also considering gender-related patterns of treatment response.

## Supplementary Information

**Additional file 1.**

## Data Availability

The datasets used and/or analyzed during the current study are available from the corresponding author on reasonable request.

## References

[1] Luime J, Koes B, Hendriksen I, Burdorf A, Verhagen A, Miedema H, Verhaar J (2004). Prevalence and incidence of shoulder pain in the general population; a systematic review. Scand J Rheumatol.

[2] Greving K, Dorrestijn O, Winters JC, Groenhof F, Van Der Meer K, Stevens M, et al. Incidence, prevalence, and consultation rates of shoulder complaints in general practice. 2012;41:150–5. 10.3109/03009742.2011.605390. 10.3109/03009742.2011.605390 21936616

[3] Littlewood C, May S, Walters S. Epidemiology of Rotator Cuff Tendinopathy: A Systematic Review. 2017;5:256–65. 10.1111/SAE.12028.

[4] Lewis J, Mccreesh K, Roy JS, Ginn K (2015). Rotator cuff tendinopathy: navigating the diagnosis-management conundrum. J Orthop Sports Phys Ther.

[5] Pellegrino R, Di Iorio A, Del Prete CM, Barassi G, Paolucci T, Tognolo L, Fiore P, Santamato A (2022). Efficacy of ultrasound-guided percutaneous lavage and biocompatible electrical Neurostimulation, in calcific rotator cuff tendinopathy and shoulder pain, a prospective pilot study. Int J Environ Res Public Health.

[6] Lewis JS (2010). Rotator cuff tendinopathy: a model for the continuum of pathology and related management. Br J Sports Med.

[7] Fama G, Tagliapietra J, Belluzzi E, Pozzuoli A, Biz C, Ruggieri P (2021). Mid-term outcomes after arthroscopic “tear completion repair” of partial thickness rotator cuff tears. Medicina (Kaunas).

[8] Cigolotti A, Biz C, Lerjefors E, de Iudicibus G, Belluzzi E, Ruggieri P (2020). Medium- to long-term clinical and functional outcomes of isolated and combined subscapularis tears repaired arthroscopically. Arch Med Sci.

[9] Modena DAO, Soares CD, Candido EC, Chaim FDM, Cazzo E, Chaim EA (2022). Effect of extracorporeal shock waves on inflammation and angiogenesis of integumentary tissue in obese individuals: stimulating repair and regeneration. Lasers Med Sci.

[10] van der Worp H, van den Akker-Scheek I, van Schie H, Zwerver J (2013). ESWT for tendinopathy: technology and clinical implications. Knee Surg Sports Traumatol Arthrosc.

[11] Agostini F, Mangone M, Finamore N, Di Nicola M, Papa F, Alessio G, Vetrugno L, Chiaramonte A, Cimbri G, Bernetti A (2022). The Efficacy of Instrumental Physical Therapy through Extracorporeal Shock Wave Therapy in the Treatment of Plantar Fasciitis: An Umbrella Review. Appl. Sci.

[12] Surace SJ, Deitch J, Johnston RV, Buchbinder R. Shock wave therapy for rotator cuff disease with or without calcification. Cochrane Database Syst Rev. 2020;2020. 10.1002/14651858.CD008962.PUB2. 10.1002/14651858.CD008962.pub2 PMC7059880 32128761

[13] Crimaldi S, Liguori S, Tamburrino P, Moretti A, Paoletta M, Toro G, Iolascon G (2021). The Role of Hyaluronic Acid in Sport-Related Tendinopathies: A Narrative Review. Med.

[14] Roerdink RL, Dietvorst M, Zwaard BVD, van der Worp H, Zwerver J (2017). Complications of extracorporeal shockwave therapy in plantar fasciitis: systematic review. Int J Surg.

[15] Auersperg V, Trieb K (2020). Extracorporeal shock wave therapy: an update. EFORT Open Rev.

[16] Pellegrino R, Brindisino F, Barassi G, Sparvieri E, Di Iorio A, De Sire A, et al. Combined ultrasound guided peritendinous hyaluronic acid (500-730 Kda) injection with extracorporeal shock waves therapy vs. extracorporeal shock waves therapy-only in the treatment of shoulder pain due to rotator cuff tendinopathy. A randomized clinical trial. J Sports Med Phys Fitness. 2022. 10.23736/S0022-4707.22.13924-1. 10.23736/S0022-4707.22.13924-1 35686864

[17] Salamanna F, Frizziero A, Pagani S, Giavaresi G, Curzi D, Falcieri E, Marini M, Abruzzo PM, Martini L, Fini M (2015). Metabolic and cytoprotective effects of in vivo peri-patellar hyaluronic acid injections in cultured tenocytes. Connect Tissue Res.

[18] Razmjou H, Davis AM, Jaglal SB, Holtby R, Richards RR. Disability and satisfaction after Rotator Cuff decompression or repair: a sex and gender analysis. 2011. 10.1186/1471-2474-12-66. 10.1186/1471-2474-12-66 PMC3083386 21457534

[19] Oh JH, Kim SH, Ji HM, Jo KH, Bin SW, Gong HS (2009). Prognostic factors affecting anatomic outcome of rotator cuff repair and correlation with functional outcome. Arthroscopy.

[20] Charousset C, Grimberg J, Duranthon LD, Bellaïche L, Petrover D, Kalra K (2008). The time for functional recovery after arthroscopic rotator cuff repair: correlation with tendon healing controlled by computed tomography arthrography. Arthroscopy.

[21] Vasilopoulos T, Wardhan R, Rashidi P, Fillingim RB, Wallace MR, Crispen PL, Parvataneni HK, Prieto HA, Machuca TN, Hughes SJ (2021). Patient and procedural determinants of postoperative pain trajectories. Anesthesiology.

[22] Pavic R, Margetic P, Bensic M, Brnadic RL. Diagnostic value of US, MR and MR arthrography in shoulder instability. Injury. 2013;44(Suppl 3). 10.1016/S0020-1383(13)70194-3. 10.1016/S0020-1383(13)70194-3 24060014

[23] MacDermid JC, Solomon P, Prkachin K (2006). The shoulder pain and disability index demonstrates factor, construct and longitudinal validity. BMC Musculoskelet Disord.

[24] Vascellari A, Venturin D, Ramponi C, Ben G, Poser A, Rossi A, Coletti N (2018). Psychometric properties of three different scales for subjective evaluation of shoulder pain and dysfunction in Italian patients after shoulder surgery for anterior instability. J Shoulder Elb Surg.

[25] Roy JS, Macdermid JC, Woodhouse LJ (2009). Measuring shoulder function: a systematic review of four questionnaires. Arthritis Care Res (Hoboken).

[26] Dubé M-O, Arel J, Paquette P, Roy J-S, Desmeules F, Gagnon DH (2022). Co-creation of an exercise inventory to improve scapular stabilization and control among individuals with rotator cuff-related shoulder pain: a survey-based study amongst physiotherapists. Arch Physiother.

[27] Singer JD. Using SAS PROC MIXED to fit multilevel models, hierarchical models, and individual growth models. J Educ Behav Stat. 1998. 10.3102/10769986023004323.

[28] Oliva F, Piccirilli E, Bossa M, Giai Via A, Colombo A, Chillemi C, Gasparre G, Pellicciari L, Franceschetti E, Rugiero C (2016). I.S.Mu.L.t - rotator cuff tears guidelines. Muscles Ligaments Tendons J.

[29] Sengar DPS, McKendry RJ, Uhthoff HK (1987). Increased frequency of HLA-A1 in calcifying tendinitis. Tissue Antigens.

[30] Sawaguchi N, Majima T, Iwasaki N, Funakoshi T, Shimode K, Onodera T, et al. Extracellular Matrix Modulates Expression of Cell-Surface Proteoglycan Genes in Fibroblasts. 2009;47:141–8. 10.1080/03008200600685459. 10.1080/03008200600685459 16753807

[31] Oliva F, Marsilio E, Asparago G, Frizziero A, Berardi AC, Maffulli N. The impact of hyaluronic acid on tendon physiology and its clinical application in tendinopathies. Cells. 2021;10. 10.3390/CELLS10113081. 10.3390/cells10113081 PMC8625461 34831304

[32] Sikes KJ, Renner K, Li J, Grande-Allen KJ, Connell JP, Cali V, Midura RJ, Sandy JD, Plaas A, Wang VM (2018). Knockout of hyaluronan synthase 1, but not 3, impairs formation of the retrocalcaneal bursa. J Orthop Res.

[33] Frizziero A, Vittadini F, Barazzuol M, Gasparre G, Finotti P, Meneghini A, Maffulli N, Masiero S (2017). Extracorporeal shockwaves therapy versus hyaluronic acid injection for the treatment of painful non-calcific rotator cuff tendinopathies: preliminary results. J. Sports Med. Phys. Fitness.

[34] Fogli M, Giordan N, Mazzoni G (2017). Efficacy and safety of hyaluronic acid (500-730kDa) ultrasound-guided injections on painful tendinopathies: a prospective, open label, clinical study. Muscles. Ligaments Tendons J..

[35] Galasso O, Amelio E, Riccelli DA, Gasparini G (2012). Short-term outcomes of extracorporeal shock wave therapy for the treatment of chronic non-calcific tendinopathy of the supraspinatus: a double-blind, randomized, placebo-controlled trial. BMC Musculoskelet Disord.

[36] Reilly JM, Bluman E, Tenforde AS (2018). Effect of shockwave treatment for Management of Upper and Lower Extremity Musculoskeletal Conditions: a narrative review. PM&R.

[37] Jeon JH, Jung YJ, Lee JY, Choi JS, Mun JH, Park WY, Seo CH, Jang KU (2012). The effect of extracorporeal shock wave therapy on myofascial pain syndrome. Ann Rehabil Med.

[38] Notarnicola A, Maccagnano G, Tafuri S, Fiore A, Margiotta C, Pesce V, Moretti B (2016). Prognostic factors of extracorporeal shock wave therapy for tendinopathies. Musculoskelet Surg.

[39] Alabas OA, Tashani OA, Tabasam G, Johnson MI (2012). Gender role affects experimental pain responses: a systematic review with meta-analysis. Eur J Pain.

