# Supplementary material for: Effectiveness of combined extracorporeal shock-wave therapy and hyaluronic acid injections for patients with shoulder pain due to rotator cuff tendinopathy: a person-centered approach with a focus on gender differences to treatment response
Source: BMC Musculoskelet Disord. 2022 Sep 15;23:863. doi: 10.1186/s12891-022-05819-3 (PMC9479346; doi:10.1186/s12891-022-05819-3)
Supplement: Supplementary file 1 — Additional file 1. [file 12891_2022_5819_MOESM1_ESM.docx]

Supplementary material Table 1: Mixed Linear Model; SPADI variation according to time of the study, only in the female patients group. Baseline assessment was the time-reference.

|  | **Estimate** | **S.E.** | **Pr > \|t\|** |
| --- | --- | --- | --- |
| Intercept | 33.98 | 13.04 | 0.01 |
| Time Follow-up 2 | -14.35 | 2.60 | <0.001 |
| Time Follow-up 1 | -13.71 | 2.59 | <0.001 |
| Baseline (reference group) |  |  |  |
| Treatment E+Hy | 3.99 | 4.17 | 0.35 |
| Treatment ESWT-al (reference group) |  |  |  |
| Time Follow-up 2 Treatment E+Hy | -19.45 | 3.35 | <0.001 |
| Time Follow-up 2 Treatment ESWT-al (reference group) |  |  |  |
| Time Follow-up 1 Treatment E+Hy | -15.21 | 3.34 | <0.001 |
| Time Follow-up 1 Treatment ESWT-al (reference group) |  |  |  |
| Shoulder pain side right | -0.70 | 3.47 | 0.84 |
| Shoulder pain side left (reference group) |  |  |  |
| Age (yy) | 0.19 | 0.26 | 0.45 |

Supplementary material Table 2: Mixed Linear Model; NRS variation according to time of the study, only in the female patients group. Baseline assessment was the time-reference.

|  | **Estimate** | **S.E.** | **Pr > \|t\|** |
| --- | --- | --- | --- |
| Intercept | 5.37 | 1.30 | <0.001 |
| Time Follow-up 2 | -1.17 | 0.29 | <0.001 |
| Time Follow-up 1 | -1.42 | 0.30 | <0.001 |
| Baseline (reference group) |  |  |  |
| Treatment E+Hy | -0.40 | 0.43 | 0.36 |
| Treatment ESWT-al (reference group) |  |  |  |
| Time Follow-up 2 Treatment E+Hy | -3.02 | 0.38 | <0.001 |
| Time Follow-up 2 Treatment ESWT-al (reference group) |  |  |  |
| Time Follow-up 1 Treatment E+Hy | -1.60 | 0.38 | <0.001 |
| Time Follow-up 1 Treatment ESWT-al (reference group) |  |  |  |
| Shoulder pain side right | -0.45 | 0.35 | 0.21 |
| Shoulder pain side left (reference group) |  |  |  |
| Age (yy) | 0.03 | 0.02 | 0.28 |
